# Quality of Life Among Patients With Ductal Carcinoma In Situ

**DOI:** 10.1001/jamanetworkopen.2025.18887

**Published:** 2025-07-03

**Authors:** Victoria J. Dunsmore, Bradley S. Snyder, Ilana F. Gareen, Constance D. Lehman, Seema A. Khan, Justin Romanoff, Constantine Gatsonis, Ralph L. Corsetti, Habib Rahbar, Derrick W. Spell, Linda K. Han, John R. Bumberry, Kathy D. Miller, Joseph A. Sparano, Christopher Comstock, Elyse Park, Lynne I. Wagner, Ruth C. Carlos

**Affiliations:** 1 University of North Carolina at Chapel Hill; 2 Center for Biostatistics and Health Data Science, Brown University School of Public Health, Providence, Rhode Island; 3 Center for Biostatistics and Health Data Science, Department of Epidemiology, Brown University School of Public Health, Providence, Rhode Island; 4 Massachusetts General Hospital, Boston; 5 Northwestern University, Chicago, Illinois; 6 Center for Biostatistics and Health Data Science, Department of Biostatistics, Brown University School of Public Health, Providence, Rhode Island; 7 Tulane University School of Medicine, New Orleans, Louisiana; 8 University of Washington, Seattle; 9 Gulf South NCORP, New Orleans, Louisiana; 10 Indiana University, Bloomington; 11 Mercy Hospital Springfield, Springfield, Missouri; 12 Montefiore Medical Center-Weiler Hospital, Bronx, New York; 13 Memorial Sloan Kettering Cancer Center, New York, New York; 14 Columbia University, New York, New York

## Abstract

**Importance:**

Limited longitudinal data exist regarding health-related quality of life (HRQL) following surgery for ductal carcinoma in situ (DCIS) breast cancer.

**Objective:**

To assess individual- and neighborhood-level factors associated with longitudinal trajectories of mental and physical HRQL among individuals with DCIS eligible for breast conservation surgery.

**Design, Setting, and Participants:**

This cohort study was an ancillary to a prospective, nonrandomized clinical trial of women with DCIS breast cancer between March 2015 and April 2016 at 75 US institutions, community practices, and academic centers coordinated by the Eastern Cooperative Oncology Group–American College of Radiology Imaging Network (ECOG-ACRIN) Cancer Research Group (E4112). Eligible participants were recently diagnosed with unilateral DCIS who were eligible for wide local excision (WLE) and had a diagnostic mammogram within 3 months of study registration. Patient-reported outcome (PRO) questionnaires were administered to participants at the time of registration, after surgeon consultation but presurgery, at the first postoperative visit, 12 months postsurgery, and 24 months postsurgery. Self-reported social determinants of health (SDOH) and clinical history and surgery received were also collected. Data were analyzed from June 2024 to November 2024.

**Main Outcomes and Measures:**

PRO measures assessing patient knowledge of DCIS and perception of being informed; HRQL, measured using the 10-item Patient-Reported Outcomes Measurement Information System with mental and physical health subscales.

**Results:**

Among the 296 women, the median age at enrollment was 60 years (range, 34-87 years) (11 Asian [4%], 41 Black [14%], 229 White [77%]); 147 participants (50%) reported at least 1 family member with breast cancer. The majority of participants had non-Hispanic ethnicity (280 [95%]), had private insurance (227 [77%]), resided in areas of low-to-moderate deprivation (ADI median, 44 [range, 1-99]), and received a single WLE (185 [63%]). Participants exhibited good knowledge of DCIS (median, 80 [range, 20-100]) and a high perception of being informed (median, 10 [range, 3-10]). Being American Indian or Alaska Native, Asian, Black, multiracial, or not reporting race (χ^2^ = 6.8 [*df*, 2]; *P* = .03), having insurance other than private (χ^2^ = 14.3 [*df*, 2]; *P* < .001), and having more than 1 surgery (χ^2^ = 12.3 [*df*, 4]; *P* = .02) were associated with decreases in mental health at 24 months compared with baseline. Having private insurance and a low perception of being informed of one’s treatment were associated with increases in physical health at 2 years.

**Conclusions and Relevance:**

In this cohort study of women with DCIS, in the 24 months following diagnosis, nonprivate insurance, minoritized race, and number of surgeries received were associated with reduced mental HRQL. Nonprivate insurance was also associated with reduced physical HRQL.

**Trial Registration:**

ClinicalTrials.gov Identifier: NCT02352883

## Key Points

**Question:**

Are clinical or social determinants of health factors associated with health-related quality of life in the 2-year period following surgery for ductal carcinoma in situ (DCIS) breast cancer?

**Findings:**

In this cohort study including 296 patients with DCIS, minoritized race (including American Indian or Alaska Native, Asian, Black, multiracial, and unknown or not reported) and receiving more than 1 surgery were associated with worsening long-term mental health, and nonprivate insurance was associated with worsening of both mental and physical health. Non-White women who received more than 1 surgery experienced a sustained decline in mental health substantially larger than the defined minimal important change.

**Meaning:**

Interventions aimed at improving mental health outcomes for at-risk DCIS patients are a crucial next step.

## Teaser

This cohort study and secondary analysis of a nonrandomized clinical trial examines health-related quality of life by race following surgery for ductal carcinoma in situ breast cancer.

## Introduction

Ductal carcinoma in situ (DCIS) is a nonobligate precursor breast cancer accounting for approximately 20% to 25% of newly diagnosed lesions that are commonly classified as malignant.^1,2^ Rates of DCIS diagnosis have risen significantly with widespread screening mammography.^1,3^ DCIS, often referred to as preinvasive or stage 0 cancer, is considered a key precursor to invasive breast cancer (IBC),^4^ with high-grade subtypes more likely to progress to IBC if left untreated.^4^ The goal of treatment in DCIS is to reduce the risk of this progression. Surgical treatment options for DCIS vary and include wide local excision (WLE), with or without radiation, as well as mastectomy.^5,6^ Survival following these treatments is high, with 10-year disease-specific survival exceeding 98%,^5^ but patient choice in receiving these treatments varies. We previously demonstrated that the process of treatment decision-making in DCIS is dynamic and influenced by surgeon recommendation, self-reported importance of keeping one’s breast, and cancer worry.^7^

What is less known is how treatment impacts long-term health-related quality of life (HRQL) in the survivorship period for women with newly diagnosed DCIS. A 2020 review showed that HRQL among women with DCIS is comparable with women with IBC, and differences in HRQL have been related to surgery type.^8^ When examining social determinants of health (SDOH) among DCIS patients, authors found no differences in HRQL by age, race, or neighborhood deprivation index.^9^ When examining patient-clinician dynamics, 1 study found that shared decision-making was positively related to HRQL in cancer.^10^ However, previous work across SDOH and shared decision-making has predominantly focused on cross-sectional assessments of individuals across the survivorship spectrum. Furthermore, the limited longitudinal information on HRQL is often available only for women with IBC.^11^ Uniquely missing are assessments of longitudinal trajectories of HRQL and factors that influence these trajectories in newly diagnosed DCIS. In addition, most work in HRQL using the Patient-Reported Outcomes Measurement Information System (PROMIS) measure has focused only on mental health outcomes rather than separate physical and mental health outcomes among people with cancer.^12^ As a result, previous research in HRQL outcomes has often lacked the nuanced understanding of factors that may differentially impact physical and mental health. To fill this gap, we assessed the longitudinal trajectories of mental and physical HRQL and individual- and neighborhood-level factors associated with these trajectories among a prospective cohort of women with newly diagnosed DCIS participating in the Eastern Cooperative Oncology Group–American College of Radiology Imaging Network (ECOG-ACRIN) Cancer Research Group (E4112) nonrandomized clinical trial.^13^

## Methods

The study was approved by the National Cancer Institute Division of Cancer Prevention, the local institutional review board at each participating site, and the Brown University institutional review board. Written informed consent was obtained from all participants (NCT02352883). Reporting of this study follows the Strengthening of the Reporting of Observational Studies in Epidemiology (STROBE) reporting guideline for cohort studies.

### Data and Sample

**Figure 1.**
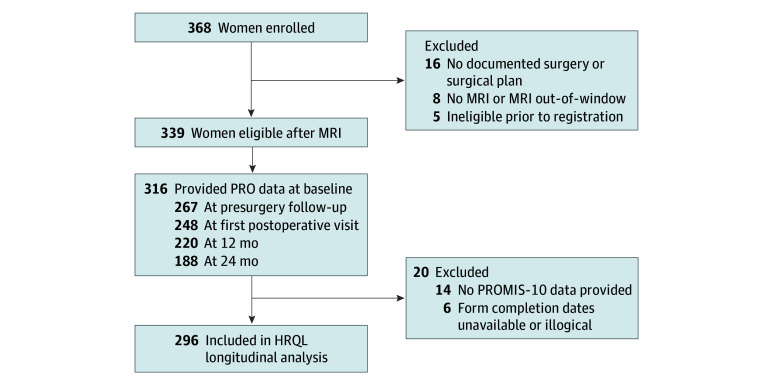
Study Flowchart HRQL indicates health-related quality of life; MRI, magnetic resonance imaging; PRO, patient-reported outcome; PROMIS-10, 10-Item Patient Reported Outcomes Measurement Information System.

This was an ancillary study to a prospective, nonrandomized clinical trial coordinated by the ECOG-ACRIN Cancer Research Group (E4112) that enrolled women with unilateral DCIS. Primary results, recruitment methods, data collection processes, and participating institutions have been previously described.^7,13^ Patient-reported outcome (PRO) questionnaires were scheduled to be provided to participants at the time of registration, after surgeon consultation but presurgery, at the first postoperative visit, 12 months postsurgery, and 24 months postsurgery (Figure 1). See eMethods in Supplement 1 for details on PRO data collection.

### Covariates

#### Clinical Factors

Surgery received was categorized as initial WLE as the sole surgery, initial mastectomy as the sole surgery, or more than 1 surgery. The latter category included women who underwent multiple WLEs to ensure clear margins (surgical margin of less than 2 mm or evidence of microinvasive or invasive carcinoma) and women who converted to mastectomy after WLE. Patients self-reported whether they had a family history of breast cancer.

#### SDOH Factors

SDOH collected at baseline included age, race, and ethnicity. Race and ethnicity were self-reported. Race categories included American Indian or Alaska Native, Asian, Black, White, multiple races, and not reported or unknown. Given the limited number of American Indian or Alaska Native, Asian, Black, and multiracial individuals, we grouped race into 2 categories (White and non-White). Additional covariates included insurance status and area deprivation index (ADI). The ADI is a composite measure that ranks neighborhoods by socioeconomic disadvantage and includes covariates such as income, educational level, and employment at the census block level.^14^ ADI scores range from 1 as the lowest to 100 as the highest level of disadvantage.^14^

#### PRO Questionnaires

We adapted items from the Breast Cancer Surgery Decision Quality Instrument developed by Sepucha et al^15^ to examine patient knowledge of DCIS and perception of being informed.^16^ Patient knowledge regarding DCIS was measured at the first postoperative visit using 5 multiple-choice knowledge items. Knowledge score represented the proportion of questions answered correctly (range, 0% to 100%). Perception of being informed was collected at presurgery consultation using 1 item, “On a scale from 0 to 10, where 10 means extremely well informed and 0 means not informed at all, how informed did you feel about surgical options for breast cancer?” (range, 0-10). All relevant PROs are contained in eMethods in Supplement 1.

### Outcomes

Mental and physical HRQL was assessed at baseline, first postoperative visit, 12 months postsurgery, and 24 months postsurgery using the Patient Reported Outcomes Measurement Information System 10-item questionnaire (PROMIS-10) for global physical and mental health.^17^ All PROMIS-10 survey items were assessed using a 5-point Likert scale, except for the average pain rating, which used a 10-point Likert scale. Summed raw scores were converted into physical and mental T scores per PROMIS-10 published criteria (physical T score range, 16.2-67.7; mental T score range, 21.2-67.6),^18^ which served as the HRQL outcomes of interest.

### Statistical Analysis

This ancillary study conducted longitudinal data analyses to assess the association between prespecified covariates of interest and mental and physical HRQL for women enrolled in E4112. Based on prior research, we defined the minimal important change (MIC) in HRQL as a 2-point difference on the PROMIS-10 T score scale.^18,19^ Given the longitudinal nature of the data, associations with covariates were assessed using linear mixed models. Both univariable and multivariable models were fit. Covariate categorization and details on the statistical models are provided in eMethods in Supplement 1.

Data were analyzed from June to November 2024 using SAS version 9.4 software (SAS Institute) and R version 4.3.1 software (R Project for Statistical Computing). All reported *P* values are 2-sided, with the significance threshold set to .05. Because this ancillary study’s aim was secondary to the main trial, analyses were considered exploratory and hypothesis-generating, so adjustment for multiplicity of inference was not performed.

## Results

### Participant Characteristics

A total of 296 women were analyzed longitudinally (Figure 1). The median age at enrollment was 60 years (range, 34-87 years), and 147 (50%) reported at least 1 family member with breast cancer (Table 1). Most participants were White (229 [77%]), with the non-White category totaling 67 respondents (23%) (2 American Indian or Alaska Native [3%], 11 Asian [16%], 41 Black [61%], 1 multiple race [1%], 12 not reported or unknown [18%]). The majority of participants were of non-Hispanic ethnicity (280 [95%]), had private insurance (227 [77%]), resided in areas of low-to-moderate deprivation (ADI median, 44 [range, 1-99]), and received a single WLE (185 [63%]). Regarding patient-reported outcomes, women preferred shared decision-making (211 [71%]) and exhibited good patient knowledge of DCIS (median, 80 [range, 20-100]) and a high perception of being informed (median, 10 [range, 3-10]). The mean (SD) for baseline PROMIS-10 mental and physical T scores for the entire cohort were 51.5 (6.7) and 52.0 (8.1), respectively (Table 1).

**Table 1.** Distributional Summaries for PROMIS-10 Mental and Physical T Scores and Prespecified Covariates

Covariates	Women, No. (%)	
	Eligible with MRI performed and known final surgery status (n = 339)	HRQL longitudinal analysis set (n = 296)
Social determinants of health covariates		
Age, median (range), y	60 (34-87)	60 (34-87)
Race		
Non-White^a^	77 (23)	67 (23)
White	262 (77)	229 (77)
Ethnicity		
Hispanic	21 (6)	16 (5)
Non-Hispanic	318 (94)	280 (95)
Insurance status		
Private	261 (77)	227 (77)
Other^b^	78 (23)	69 (23)
ADI, median (range)	44 (1-100)	44 (1-99)
Clinical covariates		
Family history of breast cancer	163 (48)	147 (50)
Type of surgery		
1 WLE	215 (63)	185 (63)
Mastectomy	54 (16)	47 (16)
>1 Surgery	70 (21)	64 (22)
PRO covariates		
Patient knowledge, median (range)	NA	80 (20-100)
Perception of being informed, median (range)	NA	10 (3-10)
Cancer worry, median (range)	NA	2.3 (1-4)
Decision autonomy preference		
Surgeon	NA	24 (8)
Shared	NA	211 (71)
Patient	NA	61 (21)
Importance of keeping the breast, median (range)	NA	7 (0-10)
Importance of removing the breast, median (range)	NA	5 (0-10)
Importance of avoiding radiation, median (range)	NA	6 (0-10)
Importance of sex life, median (range)	NA	5 (0-10)
HRQL outcomes		
PROMIS-10 mental T score, mean (SD)		
Baseline	NA	51.5 (6.7)
Postoperative follow-up	NA	51.3 (7.9)
12 months	NA	51.4 (7.5)
24 months	NA	52.0 (7.9)
PROMIS-10 physical T score, mean (SD)		
Baseline	NA	52.0 (8.1)
Postoperative follow-up	NA	50.3 (8.1)
12 months	NA	51.4 (8.1)
24 months	NA	51.9 (8.7)

Abbreviations: ADI, Area Deprivation Index; HRQL, health-related quality of life; MRI, magnetic resonance imaging; NA, not applicable; PRO, patient-reported outcome; PROMIS-10, 10-Item Patient-Reported Outcomes Measurement Information System; WLE, wide local excision.

^a^ Breakdown of non-White race: 2 American Indian/Alaskan Native, 11 Asian, 41 Black, 1 multiple race, 7 not reported, and 5 unknown.

^b^ Breakdown of other insurance: 52 Medicare, 7 Medicaid, 2 veterans sponsored, 1 Medicaid and Medicare, 1 military sponsored, 1 self-pay, 1 no means of payment (no insurance), and 4 unknown.

Of the 229 White women in the cohort, 143 (62%) received 1 WLE, 35 (15%) received a mastectomy, and 51 (22%) received more than 1 surgery; of the 67 non-White women in the cohort, 42 (63%) received 1 WLE, 12 (18%) received a mastectomy, and 13 (19%) received more than 1 surgery. Additional information regarding breast reconstruction rates after surgery can be found in eTable 1 in Supplement 1.

Women were scheduled to complete the HRQL assessment at the baseline, first postoperative visit, 12 months postsurgery, and 24 months postsurgery time points. Approximately two-thirds of women had 3 or more assessments available for both the mental (197 of 296 [67%]) and physical T scores (191 of 296 [65%]) (maximum 4). A smaller number of women contributed T score data for only 1 time point (45 of 296 [15%] mental and 49 of 296 [17%] physical) or 2 time points (54 of 296 [18%] mental and 56 of 296 [19%] physical). The median and IQR for the timing of PRO questionnaire completion by time point were: baseline, 21 (13-35) days before surgery; first postoperative visit, 30 (16-61) days after surgery; 12 months postsurgery, 405 (385-535) days; and 24 months postsurgery, 755 (749-780) days. However, there was variability among women as to the timing of the PRO questionnaires. PROMIS-10 physical and mental T scores at all available time points are shown in eFigures 1 and 2 in Supplement 1, respectively. Additional information on data missingness by covariate can be found in eTable 2 in Supplement 1.

### Mental Health

**Figure 2.**
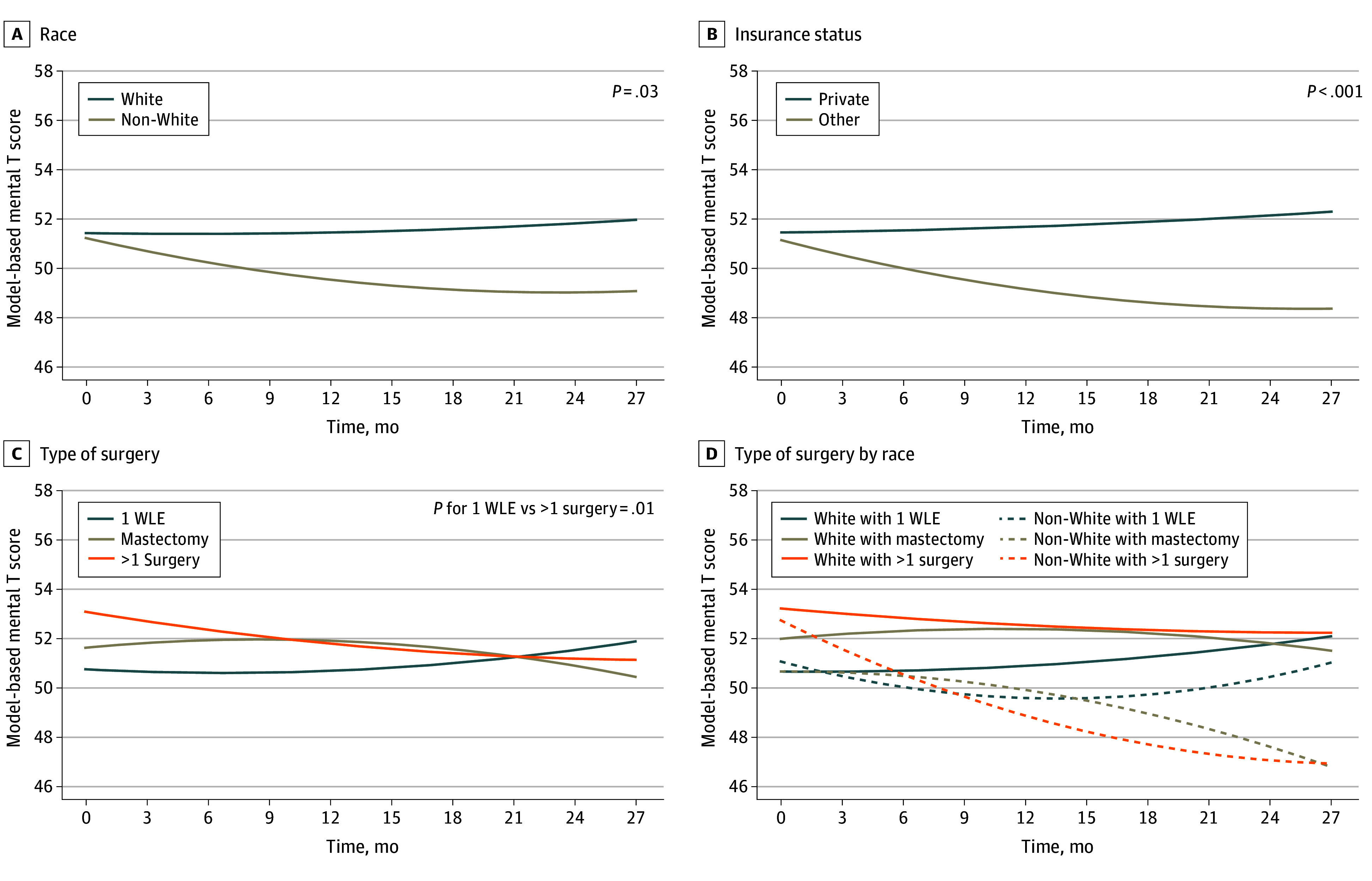
Model-Based Longitudinal Trajectories of the PROMIS-10 Mental T Score by Race, Insurance Status, and Type of Surgery D, White with 1 WLE vs non-White (American Indian or Alaska Native, Asian, Black, multiple races, and not reported or unknown) with >1 surgery, *P* < .001; White with mastectomy vs non-White with >1 surgery, *P* = .03; White with >1 surgery vs non-White with >1 surgery, *P* = .051; non-White with 1 WLE vs non-White with >1 surgery, *P* = .04. PROMIS-10 indicates 10-item Patient-Reported Outcomes Measurement Information System.

The longitudinal trajectory of mental health remained stable for the entire cohort (eFigure 3 in Supplement 1). However, differences in mental health trajectories were detected by SDOH and clinical covariates (Figure 2; Table 2). Being insured with plans other than private insurance was associated with worsening mental health over time compared with private insurance. Women with other types of insurance demonstrated a mean (SE) estimated decline through 24 months of 2.8 points (0.8) on the T score scale (*P* < .001). Race was also significantly associated with mental health trajectory, with non-White women reporting worsening mental health over time compared with White women, with a decline through 24 months of 2.2 (0.9) points (*P* = .03). Clinically, women who received more than 1 surgery reported poorer mental health over time compared with women who received 1 WLE, with a decline through 24 months of 1.9 (0.8) points (*P* = .01). The observed difference in baseline mental health for women who received different types of surgery is due to the nonrandomized nature of E4112; however, the nonparallel longitudinal trajectories for the surgery groups demonstrate this association (Figure 2). When examining the interaction between race and type of surgery, receipt of more than 1 surgery was associated with a greater decline in mental health for non-White women, who exhibited a sustained decline through 24 months of 5.7 (1.7) points. This decline was larger than that of White women with more than 1 surgery, who exhibited a more modest decrement through 24 months of 1.0 (0.9) points, although the *P* value for this comparison from the univariable model was above the significance threshold (*P* = .051). However, after adjustment for potential confounders, the longitudinal association was consistent for other insurance (comparison with private insurance: χ^2^ = 8.3 [*df*, 2]; *P* = .02) and the comparison between White women and non-White women with more than 1 surgery achieved statistical significance (χ^2^ = 8.4 [*df*, 2]; *P* = .02) (eTable 3 in Supplement 1).

**Table 2.** Differences in Longitudinal Trends in PROMIS-10 Mental and Physical T Scores by Covariate Level as Estimated by Univariable Linear Mixed Models

Covariate	Level	Differences in longitudinal trend in the PROMIS-10 mental T score		Differences in longitudinal trend in the PROMIS-10 physical T score	
		Test statistic, χ^2^ (*df*)^a^	*P* value	Test statistic, χ^2^ (*df*)^a^	*P* value
Age	Continuous (per 5-y increase)	3.4 (2)	.18	3.5 (2)	.17
Race	White (vs non-White^b^)	6.8 (2)	.03	2.4 (2)	.31
Ethnicity	Hispanic (vs non-Hispanic)	0.3 (2)	.88	0.5 (2)	.77
Insurance status	Private (vs other)	14.3 (2)	<.001	14.1 (2)	<.001
ADI (theoretical range 0-100)	Continuous (per 10-percentile increase)	5.3 (2)	.07	3.0 (2)	.22
Family history of breast cancer	Yes (vs no)	0.3 (2)	.85	0.5 (2)	.77
Type of surgery^c^	1 WLE vs 1 mastectomy vs >1 surgery	12.3 (4)	.02	2.9 (4)	.58
Patient knowledge (theoretical range 0-100)	Continuous (per 10-unit increase)	2.4 (2)	.31	3.8 (2)	.15
Perception of being informed (theoretical range 0-10)	Continuous (per 1-unit increase)	2.2 (2)	.34	7.4 (2)	.03
Cancer worry (theoretical range 1-4)	Continuous (per 1-unit increase)	0.7 (2)	.70	2.2 (2)	.33
Decision autonomy preference	Shared vs surgeon vs patient	1.9 (4)	.76	2.5 (4)	.64
Importance of keeping the breast (range 0-10)	Continuous (per 1-unit increase)	0.2 (2)	.91	0.9 (2)	.64
Importance of removing breast (theoretical range 0-10)	Continuous (per 1-unit increase)	1.3 (2)	.54	0.1 (2)	.95
Importance of avoiding radiation (theoretical range 0-10)	Continuous (per 1-unit increase)	2.1 (2)	.34	2.1 (2)	.35
Importance of sex life (theoretical range 0-10)	Continuous (per 1-unit increase)	2.8 (2)	.25	5.5 (2)	.07

Abbreviations: ADI, Area Deprivation Index; *df*, degrees of freedom; PROMIS-10, 10-Item Patient-Reported Outcomes Measurement Information System; WLE, wide local excision.

^a^ The difference in longitudinal trend was based on a joint significance test for the interaction terms between the covariate and the linear and quadratic terms for time, using the corresponding estimable contrast with appropriate degrees of freedom. A significant *P* value indicates that the estimated longitudinal curve for the PROMIS-10 mental (or physical) T score differs by covariate level and, thus, that patients at varying levels of the covariate experienced differing longitudinal trends in mental (or physical) health over time.

^b^ American Indian or Alaska Native, Asian, Black, multiple races, and not reported or unknown.

^c^ The omnibus contrast testing for equality in longitudinal trend across the 3 surgery types was statistically significant. Pairwise contrasts are as follows: 1 WLE vs >1 surgery, *P* = .01; 1 mastectomy vs >1 surgery, *P* = .37; 1 WLE vs 1 mastectomy, *P* = .09.

As an exploratory analysis, the group corresponding to more than 1 surgery (64 participants) was further subdivided into women who received multiple WLEs (53 [83%]) vs women who received a mastectomy after attempted WLE (11 [17%]), with longitudinal trajectories of mental health estimated for each subgroup (eFigure 4 in Supplement 1). Women with multiple WLEs saw a mean (SE) decline of 1.2 (0.8) points through 24 months, and women with a mastectomy following an attempted WLE saw a mean decline of 6.5 (2.1) points through 24 months.

### Physical Health

**Figure 3.**
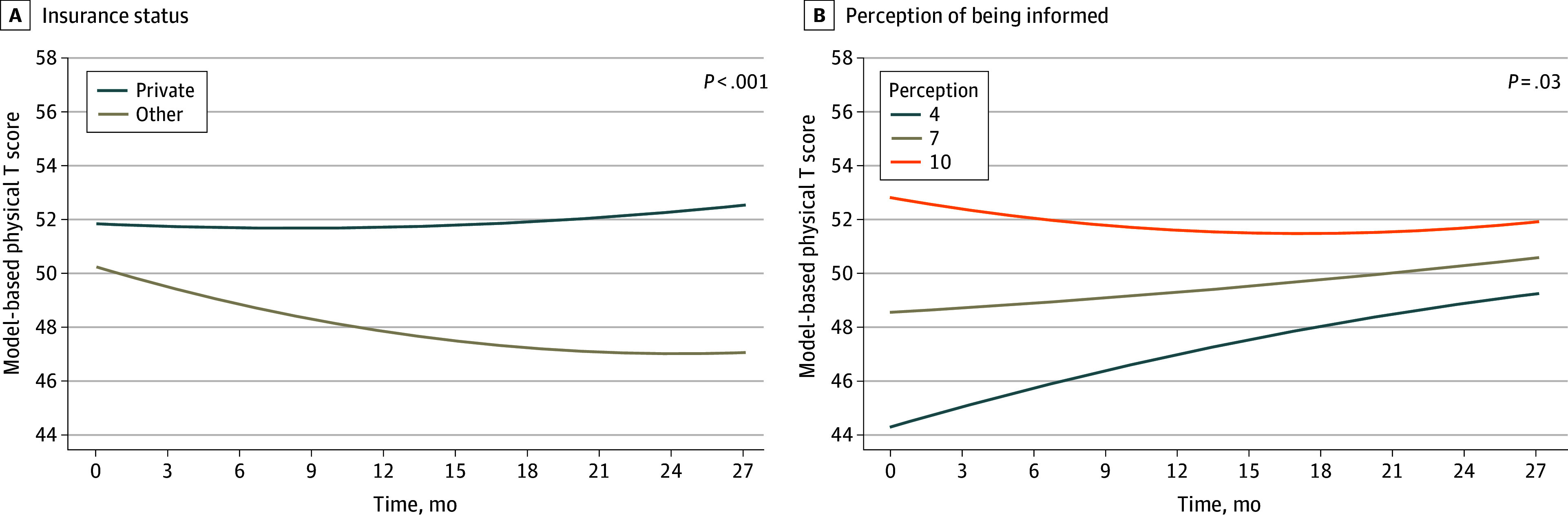
Model-Based Longitudinal Trajectories of the PROMIS-10 Physical T Score by Insurance Status and Perception of Being Informed PROMIS-10 indicates 10-item Patient-Reported Outcomes Measurement Information System.

The longitudinal trajectory of physical health remained stable for the entire cohort (eFigure 3 in Supplement 1). However, as with mental health, differences in physical health trajectories were detected by insurance status (Figure 3; Table 2). Women without private insurance reported worsening physical health over time, demonstrating a decline through 24 months of 3.2 (0.9) points on the T score scale (*P* < .001). In addition, women who reported a lower perception of being informed exhibited a larger improvement in physical health over time. There was no difference in the change in physical health over time by type of surgery. After adjustment for potential confounders, the association between other insurance and reduced physical health over 24 months remained evident (comparison with private insurance: χ^2^ = 10.0 [*df*, 2]; *P* = .007) (eTable 4 in Supplement 1).

## Discussion

In this longitudinal assessment of mental and physical health domains of HRQL, we found that non-White women with DCIS who received more than 1 surgery experienced a sustained decline in mental health. The magnitude of this decline in the mental health T score of almost 6 points 2 years after surgery was well beyond the MIC of 2. This decline was also notably worse than the corresponding decline of 1 point over 2 years experienced by White women who received more than 1 surgery. Furthermore, women with nonprivate insurance also experienced a minimal important decrement in both mental and physical health. Our study also found that while both physical and mental HRQL were associated with insurance status, physical health was also associated with decision-making factors, such as perception of being informed, while mental health was more associated with race and clinical factors including the number of surgeries.

Previous studies have shown that treatment for DCIS using mastectomy or WLE has comparable impact on physical and mental health 2 years after treatment.^20^ Our work expands on this to include the potential association of receiving more than 1 surgery, as a 2015 meta-analysis found that repeat breast surgery has been shown to occur in about 22% of patients.^21^ We found that an increased number of surgeries was related to decreased mental health 2 years after treatment among women with DCIS, with an average decline in the PROMIS-10 mental T score (1.9) very close to the MIC of 2. However, although the number of patients is small, our data suggest that the subgroup of patients who received a mastectomy after attempted WLE experienced particularly poor mental health outcomes, with an average decline of more than 6 points. The use of diagnostic technologies such as breast MRI may aid in identifying tumor extent to assist surgical planning and potentially reduce the need for repeat surgeries.^22^

In addition, we found that after controlling for age, SDOH (specifically identifying with a minoritized racial group), and having Medicare or Medicaid insurance correlated with worse mental health 2 years after treatment. Our results contrast with 2023 findings that suggest HRQL among women with DCIS did not significantly differ by race, neighborhood deprivation index, or treatment, although these findings were cross-sectional.^9^ Other studies supporting our findings show that private insurance was related to better HRQL,^23^ and that racial disparities in HRQL persist across time when examining SEER-Medicare data.^24^

Our work also assessed factors previously considered important to HRQL, namely decision-making factors, including knowledge and perception of being informed. Of these, only a patient’s perception of being informed was significantly associated with physical health in the early postoperative period. Women who rated their perception of being informed as low before surgery were more likely to experience an improvement in physical health scores. However, we would note that individuals with a low perception of being informed started the study with lower levels of physical health. Given that women with a higher perception of being informed began the study with better physical health, there may be a ceiling effect as to how much improvement these women could experience, especially given that these women had DCIS and not another type of cancer requiring more invasive or extensive treatment. Physical health scores converged 2 years after treatment across all levels of perception of being informed. Previous work has shown that physical health returns to baseline years after treatment.^8^ Our work adds nuance to this by accounting for SDOH, including lack of private insurance, which was associated with a minimal important decrease in physical health. None of the evaluated decision-making factors correlated with mental health.

### Strengths and Limitations

Study strengths include longitudinal evaluation of HRQL in a fairly large prospective cohort of women with DCIS and separate assessment of physical and mental health domains. However, there are several limitations to our work. First, our study was not randomized, so findings related to the type of surgery should be interpreted cautiously. Second, the number of non-White women who received more than 1 surgery and the number of women with mastectomy following attempted WLE were both small, so the reported findings of declines in mental health through 24 months well above the MIC for these groups, while suggestive, will require validation in a larger cohort. Rates of certain treatments tend to be related to SDOH and patient-related factors,^25,26^ which could impact mental and physical health over and above treatment received. Third, while we controlled for age, we may not have sufficiently disentangled the effect of age, comorbidity, and Medicare or Medicaid insurance status. Fourth, there were fewer survey responses from later time points compared with the number of baseline surveys completed. This is due to long-term attrition, a common barrier in longitudinal work, potentially limiting generalizability. Fifth, our data were collected from 2015 to 2018 and may not capture more recent ecological changes that may currently contribute to HQRL. Nevertheless, we believe that our data continue to reflect the general trajectory, particularly for differences between White and non-White individuals. Furthermore, in the post–COVID-19 era, we believe that there may be wider gaps in HRQL between White and non-White individuals. Lastly, due to limited numbers, we were unable to assess more granular groupings of race and ethnicity beyond the non-White grouping. Future work should recruit a larger, more diverse pool of patients, as these differences may impact HRQL.

## Conclusions

The 5-year survival rate for DCIS is exceptionally high; therefore, optimizing HRQL in this population is of paramount importance. Worryingly, mental health trajectories show sustained decrement at 2 years after surgery, particularly for non-White women, women without private insurance, and women who received more than 1 surgery. Future work may focus on a broader set of interventions tailored to at-risk groups who could be more likely to report worse mental health over time following surgery for DCIS.

## Notes

Supplement 1.
eMethods.
**eTable 1.** Breast Reconstruction Rates Among Women in the HRQL Longitudinal Analysis Set Who Received a Mastectomy **eTable 2.** Amount of Missing Data for Each Prespecified Covariate Among Women in the HRQL Longitudinal Analysis Set **eTable 3.** Adjusted Joint Significance Tests for Longitudinal Trends From a Multivariable Linear Mixed Model for the PROMIS-10 Mental T Score **eTable 4.** Adjusted Joint Significance Tests for Longitudinal Trends From a Multivariable Linear Mixed Model for the PROMIS-10 Physical T Score **eFigure 1.** Scatterplot (With Jittering) of PROMIS-10 Mental T Scores, Including All Available Time Points (T0, T2, T3, T4) **eFigure 2.** Scatterplot (With Jittering) of PROMIS-10 Physical T Scores, Including All Available Time Points (T0, T2, T3, T4) **eFigure 3.** Model-Based Longitudinal Trajectories of the PROMIS-10 Mental and Physical T Score for the Entire Study Cohort Based on Estimated Marginal Mean Values **eFigure 4.** Model-Based Longitudinal Trajectories of the PROMIS-10 Mental T Score by Type of Surgery Based on Estimated Marginal Mean Values
eReferences.

Supplement 2.
Data Sharing Statement
