# Supplementary material for: Quality of Life Among Patients With Ductal Carcinoma In Situ
Source: JAMA Netw Open. 2025 Jul 3;8(7):e2518887. doi: 10.1001/jamanetworkopen.2025.18887 (PMC12232187; doi:10.1001/jamanetworkopen.2025.18887)
Supplement: Supplement 1. — eMethods. eTable 1. Breast Reconstruction Rates Among Women in the HRQL Longitudinal Analysis Set Who Received a Mastectomy eTable 2. Amount of Missing Data for Each Prespecified Covariate Among Women in the HRQL Longitudinal Analysis Set eTable 3. Adjusted Joint Significance Tests for Longitudinal Trends From a Multivariable Linear Mixed Model for the PROMIS-10 Mental T Score eTable 4. Adjusted Joint Significance Tests for Longitudinal Trends From a Multivariable Linear Mixed Model for the PROMIS-10 Physical T Score eFigure 1. Scatterplot (With Jittering) of PROMIS-10 Mental T Scores, Including All Available Time Points (T0, T2, T3, T4) eFigure 2. Scatterplot (With Jittering) of PROMIS-10 Physical T Scores, Including All Available Time Points (T0, T2, T3, T4) eFigure 3. Model-Based Longitudinal Trajectories of the PROMIS-10 Mental and Physical T Score for the Entire Study Cohort Based on Estimated Marginal Mean Values eFigure 4. Model-Based Longitudinal Trajectories of the PROMIS-10 Mental T Score by Type of Surgery Based on Estimated Marginal Mean Values eReferences. [file jamanetwopen-e2518887-s001.pdf]

Supplemental Online Content

Dunsmore VJ, Snyder BS, Gareen IF, et al. Quality of life among patients with ductal carcinoma in situ. *JAMA Netw Open.* 2025;8(7):e2518887. doi:10.1001/jamanetworkopen.2025.18887

eMethods.

eTable 1. Breast Reconstruction Rates Among Women in the HRQL Longitudinal Analysis Set Who Received a Mastectomy

eTable 2. Amount of Missing Data for Each Prespecified Covariate Among Women in the HRQL Longitudinal Analysis Set

eTable 3. Adjusted Joint Significance Tests for Longitudinal Trends From a Multivariable Linear Mixed Model for the PROMIS-10 Mental T Score

eTable 4. Adjusted Joint Significance Tests for Longitudinal Trends From a Multivariable Linear Mixed Model for the PROMIS-10 Physical T Score

eFigure 1. Scatterplot (With Jittering) of PROMIS-10 Mental T Scores, Including All Available Time Points (T0, T2, T3, T4)

eFigure 2. Scatterplot (With Jittering) of PROMIS-10 Physical T Scores, Including All Available Time Points (T0, T2, T3, T4)

eFigure 3. Model-Based Longitudinal Trajectories of the PROMIS-10 Mental and Physical T Score for the Entire Study Cohort Based on Estimated Marginal Mean Values

eFigure 4. Model-Based Longitudinal Trajectories of the PROMIS-10 Mental T Score by Type of Surgery Based on Estimated Marginal Mean Values

eReferences.

This supplemental material has been provided by the authors to give readers additional information about their work.

© 2025 Dunsmore VJ et al. *JAMA Network Open*.

Methods

1. Data collection

The ECOG-ACRIN Outcomes and Economics Assessment Unit (OEAU) at Brown University coordinated PRO data collection. At E4112 study registration, women were offered the option of completing questionnaires using a web-based application or by mail (paper and pencil). Questionnaire administration was triggered upon the completion of study milestones, as marked by the submission of E4112 study forms.

Women electing web-based completion were invited to complete web-based questionnaires via an email prompt. We provided a study-specific toll-free telephone number to provide direct contact with the OEAU for women who had questions or needed assistance. Women who failed to complete the web-based questionnaires within the specified number of days received follow-up emails. If there was no response, OEAU attempted telephone contact.

For women preferring paper administration, mailed questionnaire packets included a letter introducing the study and the study-specific toll-free phone number for patients to reach the OEAU staff should they have questions or need assistance, together with pre-addressed, stamped envelopes for return mailing to the OEAU. If women did not complete and return the questionnaires within 10 working days of the mailing date, the OEAU attempted telephone contact.

Participants' addresses were linked to the 2020 US census data at the block group level to obtain ADI rankings. If the address lookup failed, then the full nine-digit zip code (zip+4) was used in combination with a census block group crosswalk provided by the University of Wisconsin. If the full nine-digit zip code was unavailable, then the standard five-digit zip code was used, averaging over all ratings returned. A small number of ADI rankings were based on post office (PO) boxes or only the five-digit zip code.

2. PRO items and score calculation

Patient Knowledge (5 items, 3-point scale, Sepucha et al., 2012)

- *For most women with DCIS, how much would waiting a few weeks to make a treatment decision affect their chances of survival?* [A lot, Some, A little or not at all]
- *With treatment, about how many women diagnosed with early breast cancer will eventually die of breast cancer?* [Most will die of breast cancer, About half will die of breast cancer, Most will die of something else]
- *After which treatment is it more likely that women will need to have another operation to remove more tumor cells?* [Lumpectomy, Mastectomy, Equally likely for both]
- *On average, which women with DCIS live longer?* [Women who have a mastectomy, Women who have a lumpectomy, There is no difference]
- *On average, which women have a higher chance of having cancer come back in the breast that has been treated?* [Women who have a mastectomy, Women who have a lumpectomy, There is no difference]

The correct answers were totaled, divided by 5, and then multiplied by 100 to achieve a 0 to 100 scale, with 100 representing the highest knowledge of DCIS and 0 representing the lowest knowledge of DCIS.

© 2025 Dunsmore VJ et al. *JAMA Network Open*.

Perception of Being Informed (1 item; 11-point scale) (0 (Not at all informed) to 10 (Extremely well informed))

- *"On a scale from 0 to 10, where 10 means extremely well informed and 0 means not informed at all, how informed did you feel about surgical options for breast cancer?"*

This item was treated as a semi-continuous measure ranging from 0-10.

Decision autonomy preference – Control Preferences Scale (1 item, 5-point scale; Degner et al., 1997)

- *Which of the following statements best describes how you prefer that decisions about which surgical treatment to have for your DCIS are made? Please mark ONE.* [My surgeon(s) should make the decision with little input from me; My surgeon(s) should make the decision but seriously consider my opinion; My surgeon(s) and I should make the decision together; I should make the decision after seriously considering my surgeon(s) opinion; I should make the decision with little input from my surgeon(s).]

Responses were grouped as Surgeon (My surgeon(s) should make the decision with little input from me/My surgeon(s) should make the decision but seriously consider my opinion), Shared (My surgeon(s) and I should make the decision together), and Patient (I should make the decision after seriously considering my surgeon(s) opinion/I should make the decision with little input from my surgeon(s)).

Cancer worry – Assessment of Survivor Concerns Subscale (3 items, 4-point scale; Gotay et al., 2007) (1 (not at all), 2 (a little bit), 3 (somewhat), and 4 (very much))

- *I worry about future diagnostic tests*
- *I worry about another type of cancer*
- *I worry about my cancer coming back*

The mean of the three cancer worry items (fear of diagnostic tests, new cancer diagnosis, and cancer recurrence) was calculated to obtain a semicontinuous measure ranging from 1 to 4, with higher values indicating greater levels of cancer worry.

Treatment goals and concerns (4 items; 11-point scale; Sepucha et al., 2007 & Hawley et al., 2009) (0 (Not at all important) to 10 (extremely important))

- *How important is it to you to keep your breast?*
- *How important is it to you to remove your entire breast to gain peace of mind?*
- *How important is it to you to avoid having radiation?*
- *How important is it that the type of surgery you have would not interfere with your sex life in the long term?*

Each of the 4 items was treated as a semi-continuous measure ranging from 0-10.

3. Statistical Analysis

The outcome of interest was health-related quality of life (HRQL) as measured by the PROMIS-10 mental and physical T scores collected at T0, T2, T3, and T4 (baseline, first postoperative visit, 12 months postsurgery, and 24 months postsurgery). Prespecified covariates included Social Determinants of Health (SDOH), clinical, and PRO data. SDOH covariates included age (continuous), race (White vs. non-White), ethnicity (Hispanic vs. non-Hispanic), insurance status (Private insurance vs. Other), and ADI (continuous). Clinical covariates included family history of breast cancer (Yes vs. No) and type of surgery (single WLE vs. single mastectomy vs. >1 surgery). An exploratory analysis further subdivided the >1 surgery category into multiple WLEs vs.

© 2025 Dunsmore VJ et al. *JAMA Network Open*.

mastectomy following attempted WLE. PRO covariates included patient knowledge (continuous), perception of being informed (continuous), cancer worry (continuous), decision autonomy preference (Patient vs. Shared vs. Surgeon), the importance of keeping the breast (continuous), the importance of removing the breast (continuous), the importance of avoiding radiation (continuous), and the importance of not interfering with one's sex life (continuous).

The number of available PROMIS-10 mental and physical T-scores, as well as the timing of questionnaire completion, were summarized. Distributional summaries of mental and physical T scores were reported using mean and standard deviation. Covariates were summarized using frequencies and percentages for categorical variables and medians and ranges for continuous variables.

Exploratory plots were first produced for each covariate of interest. The T score was plotted against time, with the trend by covariate level estimated using loess smoothing [1]. This was done separately for mental and physical T scores, with continuous covariates binned into reasonable categories.

Next, univariable models were fit to formally test for potential trends noted in the exploratory plots. All available PROMIS-10 T score data from time points T0, T2, T3, and T4 were modeled. We employed a linear mixed model fitted using restricted maximum likelihood estimation (REML). The linear mixed model accommodates unbalanced data (where measurement times vary across subjects), and inference remains valid under the assumption that the outcome data are missing at random (MAR) [2]. Separate models were fitted for both mental and physical T scores. Each covariate was separately examined, where fixed effects included an intercept, a linear term for time (from T0), a quadratic term for time (from T0), the covariate main effect, an interaction between the covariate and the linear term for time, and an interaction between the covariate and the quadratic term for time. The quadratic term for time allows for curvature in the longitudinal trend, and the interaction terms allow for a different longitudinal curve by covariate level. Subject-level random effects included a random intercept and a random slope. There were insufficient time points to include a subject-level random quadratic term; however, including a random slope yields variance and covariance terms in the within-subject variance-covariance matrix that vary over time, thus providing a flexible model [2].

The proposed model can simultaneously assess for differences in baseline HRQL (initial status) and differences in HRQL over time (longitudinal trends). Specifically, the significance test for the covariate main effect formally tests for differences in initial status between covariate levels (at time=0), and the joint significance test for the interaction terms, based on the corresponding estimable contrast with appropriate degrees of freedom, formally tests for differences in the longitudinal trend by covariate level. For example, for the type of surgery, the univariable linear mixed model is as follows for patient i at time point j:

$$Y_{ij} = \mathbf{B0} + \mathbf{B1}WLE_i + \mathbf{B2}Mastectomy_i + \mathbf{B3}Time_{ij} + \mathbf{B4}(Time_{ij})^2 + \mathbf{B5}Time_{ij}*WLE_i + \mathbf{B6}Time_{ij}*Mastectomy_i + \mathbf{B7}(Time_{ij})^2*WLEi + \mathbf{B8}(Time_{ij})^2*Mastectomy_i + \mathbf{b0_i} + \mathbf{b1_i}Time_{ij} + \varepsilon_{ij}$$

where WLE_i=1 if subject i received a single WLE as the sole surgery, $Mastectomy_i$=1 if subject i received mastectomy as the sole surgery, $Time_{ij}$ denotes time from T0, $(Time_{ij})^2$ denotes the square of time from T0, $\mathbf{b0_i} \sim N(0,\sigma_0^2)$ is a random intercept term, $\mathbf{b1_i} \sim N(0,\sigma_1^2)$ is a random slope term, and $\varepsilon_{ij} \sim N(0,\sigma_e^2)$ is a random error term. Coefficients **B1** and **B2** represent baseline differences in HRQL by type of surgery (here >1 surgery is the reference level). Thus, the null hypothesis of no baseline difference in HRQL between subjects with 1 WLE and subjects with >1 surgery is **Ho: B1=0**, and the null hypothesis of no baseline difference in HRQL between subjects with mastectomy and subjects with >1 surgery is **Ho: B2=0**. Likewise, the null hypothesis of no differences in longitudinal trends in HRQL by type of surgery is **Ho: B5=B6=B7=B8=0**, which can be tested using a joint chi-square test with 4 degrees of freedom.

© 2025 Dunsmore VJ et al. *JAMA Network Open*.

The above model is equivalent to the following multilevel model [3]:

Level 1: $Y_{ij} = \pi_{0i} + \pi_{1i}Time_{ij} + \pi_{2i}Time^2_{ij} + \varepsilon_{ij}$

Level 2:
(Intercept) $\pi_{0i} = \gamma_{00} + \gamma_{01}WLE_i + \gamma_{02}Mastectomy_i + \xi_{0i}$
(Slope) $\pi_{1i} = \gamma_{10} + \gamma_{11}WLE_i + \gamma_{12}Mastectomy_i + \xi_{1i}$
(Quadratic) $\pi_{2i} = \gamma_{20} + \gamma_{21}WLE_i + \gamma_{22}Mastectomy_i$

where ε_{ij} is a random error term distributed as $\varepsilon_{ij} \sim N(0,\sigma_e^2)$, and
ξ_{0i}, ξ_{1i} are random subject effects distributed as $\begin{bmatrix}\xi 0i\\ \xi 1i\end{bmatrix} \sim N(\begin{bmatrix}0\\0\end{bmatrix}, \begin{bmatrix}\sigma_0^2 & \sigma\\ \sigma & \sigma_1^2\end{bmatrix})$

After assessing the univariable models, a multivariable model was fitted, adjusting for the type of surgery, the SDOH covariates of age, race, ethnicity, and insurance status, as well as covariates showing significant differences in either initial status or longitudinal trend in HRQL.

We also fit the same linear mixed model without covariates to assess the average longitudinal trend for the entire cohort for mental and physical T scores. This provides context for the longitudinal differences seen within covariate levels. For this model, fixed effects included only an intercept, a linear term for time (from T0), and a quadratic term for time (from T0), as follows:

$$Y_{ij} = \mathbf{B0} + \mathbf{B1}Time_{ij} + \mathbf{B2}(Time_{ij})^2 + \mathbf{b0_i} + \mathbf{b1_i}Time_{ij} + \varepsilon_{ij}$$

where, again, $\mathbf{b0_i} \sim N(0,\sigma_0^2)$ is a random intercept term, $\mathbf{b1_i} \sim N(0,\sigma_1^2)$ is a random slope term, and $\varepsilon_{ij} \sim N(0,\sigma_e^2)$ is a random error term. This allows us to assess the longitudinal trend for the entire cohort, and the null hypothesis of no longitudinal change (i.e., a stable or flat trend) is **Ho: B1=B2=0**.

All linear models were fitted using PROC MIXED in SAS, and model adequacy was assessed by examining residual plots.

© 2025 Dunsmore VJ et al. *JAMA Network Open*.

Results

Table S1. Breast reconstruction rates among women in the HRQL longitudinal analysis set who received a mastectomy.

	Breast reconstruction?		
	**No**<br>**N (%)**	**Yes**<br>**N (%)**	**Total**<br>**N (%)**
**Race**			
Non-white	5<br>33%	10<br>67%	15<br>26%
White	13<br>30%	30<br>70%	43<br>74%
**Type of surgery**			
Initial mastectomy	15<br>32%	32<br>68%	47<br>81%
Attempted WLE followed by mastectomy	3<br>27%	8<br>73%	11<br>19%
**Overall**	18<br>31%	40<br>69%	58 [1]<br>100%

[1] A total of 58 women in the HRQL longitudinal analysis set received a mastectomy, of which 47 (81%) received an initial mastectomy, and 11 (19%) received a mastectomy following attempted WLE.

© 2025 Dunsmore VJ et al. *JAMA Network Open*.

Table S2. Amount of missing data for each prespecified covariate among women in the HRQL longitudinal analysis set.

	HRQL analysis set (n=296)	
	**Number (%) available**	**Number (%) missing**
**Social Determinants of Health (SDOH) covariates**		
Age	296 (100%)	0 (0%)
Race	296 (100%)	0 (0%)
Ethnicity	296 (100%)	0 (0%)
Insurance status	296 (100%)	0 (0%)
ADI	291 (98%)	5 (2%)
**Clinical covariates**		
Family history of breast cancer	296 (100%)	0 (0%)
Type of surgery	296 (100%)	0 (0%)
**Patient-reported Outcome (PRO) covariates**		
Patient knowledge	231 (78%)	65 (22%)
Perception of being informed	245 (83%)	51 (17%)
Decision Autonomy Preference	296 (100%)	0 (0%)
Cancer worry	291 (98%)	5 (2%)
Importance of keeping the breast	294 (99%)	2 (1%)
Importance of removing the breast	293 (99%)	3 (1%)
Importance of avoiding radiation	294 (99%)	2 (1%)
Importance of sex life	290 (98%)	6 (2%)

© 2025 Dunsmore VJ et al. *JAMA Network Open*.

Table S3. Adjusted joint significance tests for longitudinal trends from a multivariable linear mixed model for the PROMIS-10 mental T score.

**Adjusted joint significance test** [1]	**Test statistic** [2]	**p-value**
H_o: No difference in mental HRQL over time for Private insurance vs Other	$\chi^2 = 8.34$ (df=2)	**0.02**
H_o: No difference in mental HRQL over time for White/1 WLE vs. Non-White/>1 surgery	$\chi^2 = 12.02$ (df=2)	**0.002**
H_o: No difference in mental HRQL over time for White/Mastectomy vs. Non-White/>1 surgery	$\chi^2 = 10.48$ (df=2)	**0.005**
H_o: No difference in mental HRQL over time for White/>1 surgery vs. Non-White/>1 surgery	$\chi^2 = 8.37$ (df=2)	**0.02**

[1] The multivariable model adjusted for age, race, ethnicity, insurance status, and type of surgery, as well as covariates showing significant differences in either initial status or longitudinal trend in mental HRQL, including ADI, patient knowledge, perception of being informed, and cancer worry.

[2] The differences in longitudinal trends were based on joint significance tests for the interaction terms between the covariate and the linear and quadratic terms for time, using the corresponding estimable contrast with appropriate degrees of freedom. A significant p-value indicates that the estimated longitudinal curve for the PROMIS-10 mental T score differs by covariate level and, thus, that patients at varying levels of the covariate experienced differing longitudinal trends in mental health over time.

χ^2=chi-square test statistic; df=degrees of freedom

© 2025 Dunsmore VJ et al. *JAMA Network Open*.

Table S4. Adjusted joint significance tests for longitudinal trends from a multivariable linear mixed model for the PROMIS-10 physical T score.

**Adjusted joint significance test** [1]	**Test statistic** [2]	**p-value**
H_o: No difference in physical HRQL over time for Private insurance vs Other	10.04 (df=2)	**0.007**
H_o: No difference in physical HRQL over time among varying levels of Perception of being informed	5.74 (df=2)	0.06

[1] The multivariable model adjusted for age, race, ethnicity, insurance status, and type of surgery, as well as covariates showing significant differences in either initial status or longitudinal trend in physical HRQL, including ADI, patient knowledge, perception of being informed, cancer worry, importance of keeping the breast, and importance of sex life.

[2] The difference in longitudinal trend was based on a joint significance test for the interaction terms between the covariate and the linear and quadratic terms for time, using the corresponding estimable contrast with appropriate degrees of freedom. A significant p-value indicates that the estimated longitudinal curve for the PROMIS-10 physical T score differs by covariate level and, thus, that patients at varying levels of the covariate experienced differing longitudinal trends in physical health over time.

χ^2=chi-square test statistic; df=degrees of freedom

© 2025 Dunsmore VJ et al. *JAMA Network Open*.

Figure S1. Scatterplot (with jittering) of PROMIS-10 mental T scores, including all available time points (T0, T2, T3, T4).

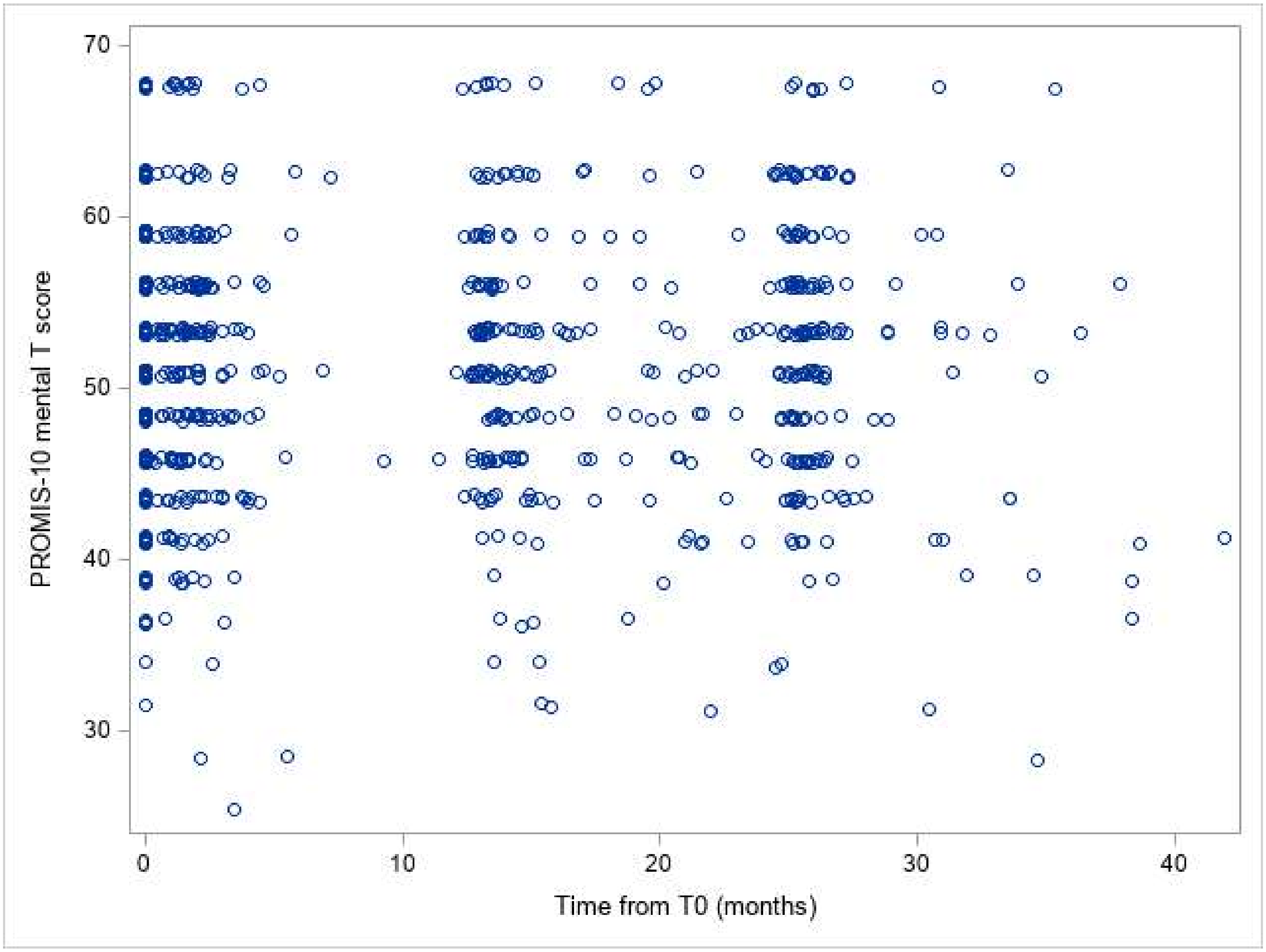

© 2025 Dunsmore VJ et al. *JAMA Network Open*.

Figure S2. Scatterplot (with jittering) of PROMIS-10 physical T scores, including all available time points (T0, T2, T3, T4).

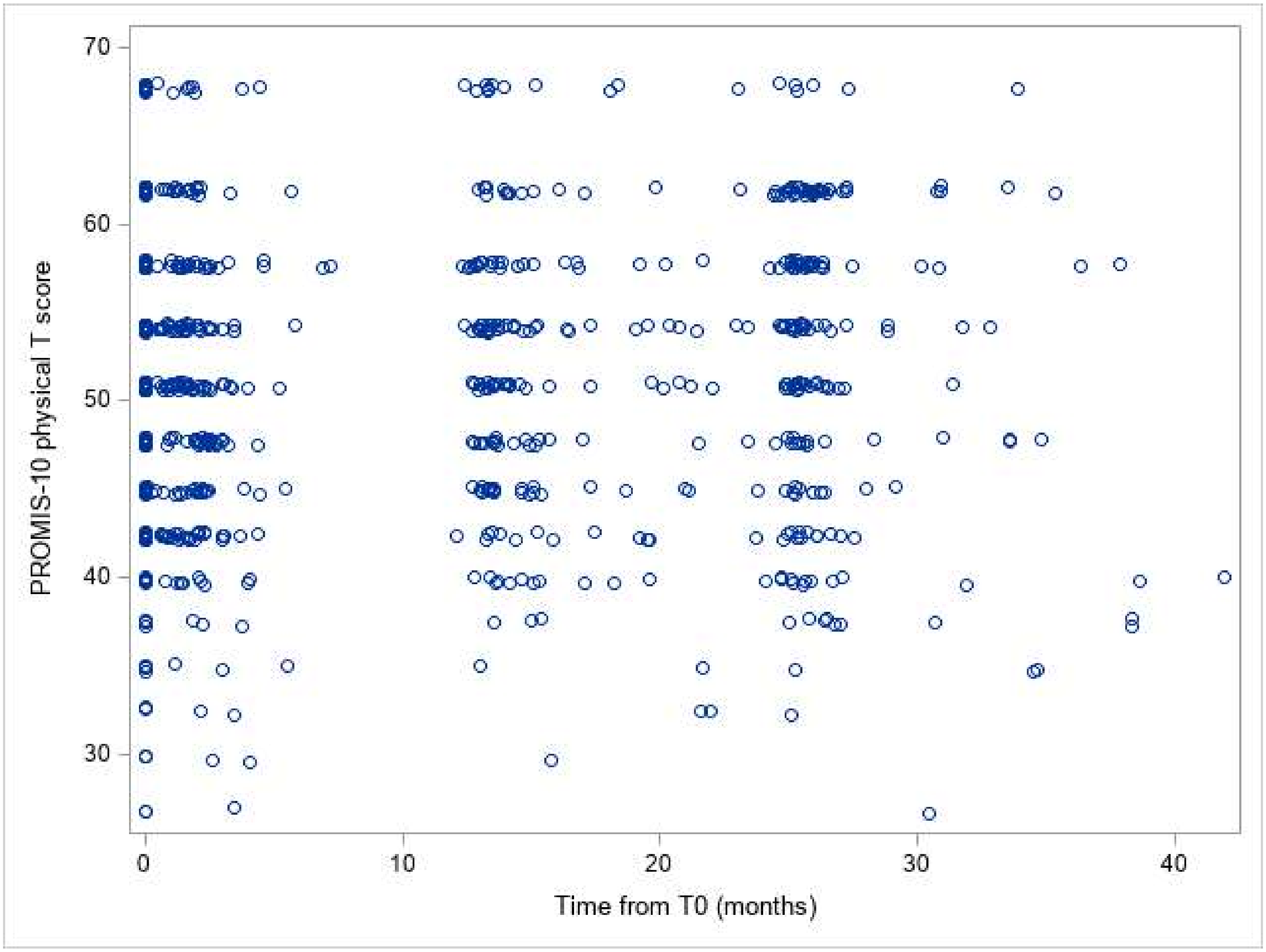

© 2025 Dunsmore VJ et al. *JAMA Network Open*.

Figure S3. Model-based longitudinal trajectories of the PROMIS-10 mental and physical T score for the entire study cohort based on estimated marginal mean values.

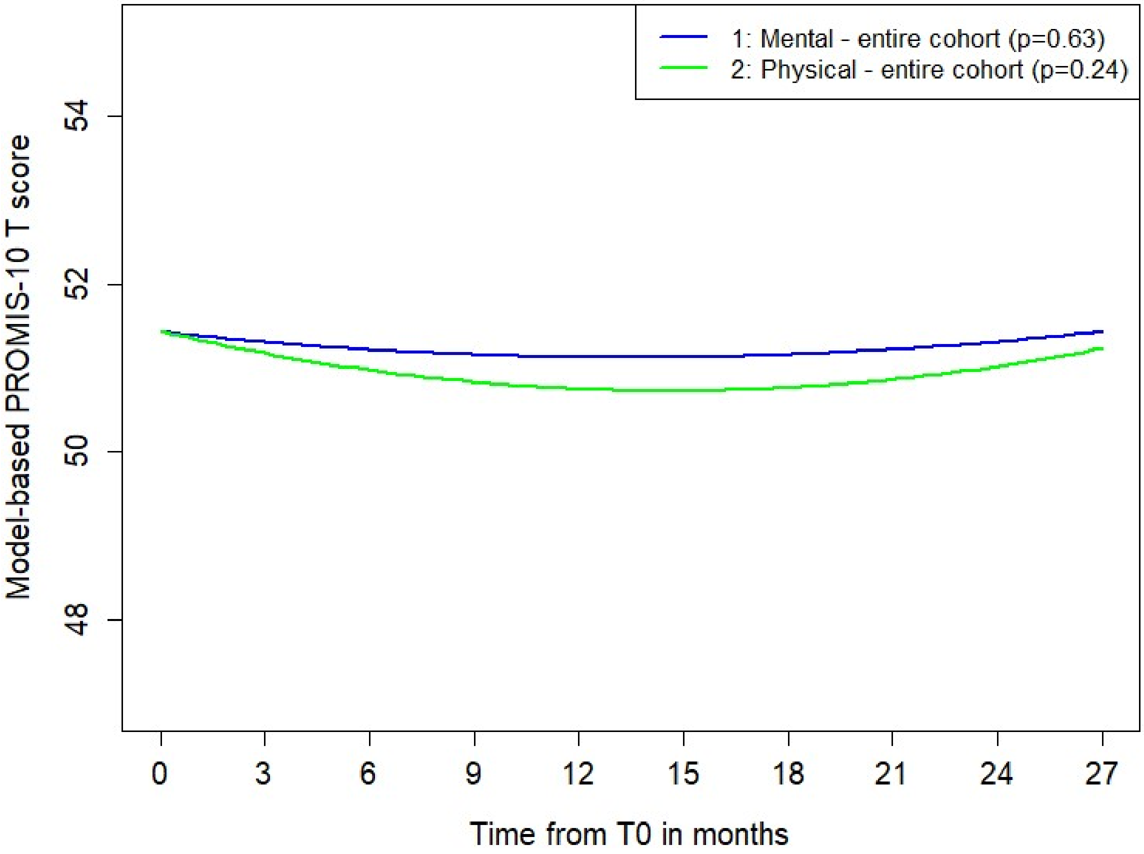

Averaged over the entire cohort, the overall trend was flat, showing no change over time for either mental (p=0.63) or physical (0.24) health.

© 2025 Dunsmore VJ et al. *JAMA Network Open*.

Figure S4. Model-based longitudinal trajectories of the PROMIS-10 mental T score by type of surgery based on estimated marginal mean values.

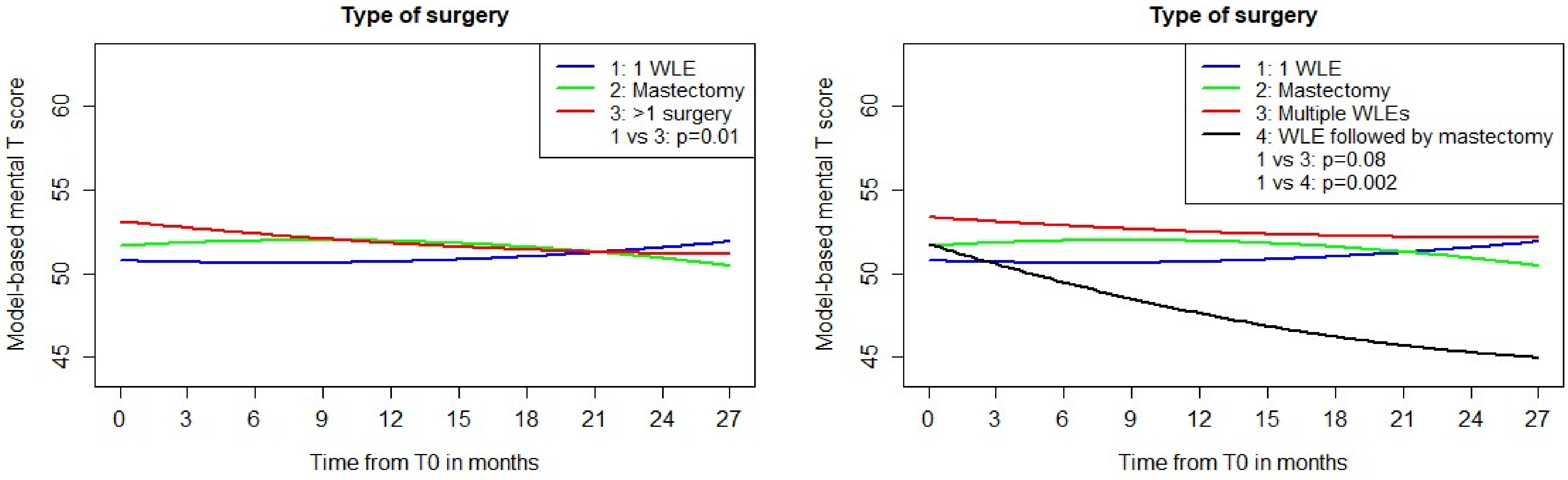

The figure in the left panel combines all women with >1 surgery and is the same as Figure 2 in the main paper. The figure in the right panel further breaks out women with >1 surgery (n=64) into those who had multiple WLEs (n=53, 83%) and those who had a mastectomy following attempted WLE (n=11, 17%).

As reported in the main paper, women who underwent more than one surgery experienced an average decline in the mental T score of 1.9 points (SE 0.77) through 24 months, which was statistically significant compared to women with 1 WLE (p=0.01). When the >1 surgery group is further broken out, women with multiple WLEs experienced an average decline of 1.2 points (SE 0.82) through 24 months, and women with a mastectomy following an attempted WLE experienced an average decline of 6.5 points (SE 2.1) through 24 months. However, it should be noted that the number of women with a mastectomy following attempted WLE is small (n=11), so this finding will require validation in larger cohorts. In addition, because outlying points can have more influence on curve-fitting when there are smaller counts, future studies may not demonstrate a difference as large as ours.

© 2025 Dunsmore VJ et al. *JAMA Network Open*.

References

1. Cleveland WS, Devlin SJ, Grosse E. "Regression by Local Fitting." Journal of Econometrics 37:87-114; 1988
2. Fitzmaurice GM, Laird NM, Ware JH. *Applied Longitudinal Analysis*. 2nd edition. John Wiley & Sons; 2011.
3. Singer JD, Willett JB. *Applied Longitudinal Data Analysis*. Oxford University Press; 2003.

© 2025 Dunsmore VJ et al. *JAMA Network Open*.
