# Supplementary material for: Quality of Life Among Patients With Ductal Carcinoma In Situ
Source: JAMA Netw Open. 2025 Jul 3;8(7):e2518887. doi: 10.1001/jamanetworkopen.2025.18887 (PMC12232187; doi:10.1001/jamanetworkopen.2025.18887)
Supplement: Supplement 2. — Data Sharing Statement [file jamanetwopen-e2518887-s002.pdf]

Data Sharing Statement

Dunsmore. Quality of Life Among Patients With Ductal Carcinoma in Situ. *JAMA Netw Open*. Published July 02, 2025. doi:10.1001/jamanetworkopen.2025.18887

Data
Data available: No

Additional Information
Explanation for why data not available: The data from the present publication will be made available by request from the ECOG-ACRIN Cancer Research Group (https://ecog-acrin.org/)
